# Progress in Surgery

**Published:** 1900-08-04

**Authors:** 


### Progress in Surgery.

### HERNIA.

(*Continued from page* 287.)

**Resection of Intestine for Gangrenous Hernia.**—J. Hutchinson, jun.,[3] recently related two successful cases of primary resection of small intestine during herniotomy at the Clinical Society of London. In the second case 10 inches of intestine had been removed by Maunsell's method. Three years later the patient died, and the specimen was obtained. It was shown at the meeting. There had not been the slightest narrowing at the site of the resection. Hutchinson remarked that he had collected from hospital records 15 examples of primary resection for gangrenous gut in which Murphy's button was used, and 16 in which suturing alone was employed. Of the former, only 1 (7 per cent.) had recovered; of the latter, 6 (40 per cent.) Besides this the fatal cases treated by suturing alone had lived twice as long, on an average, as those in which the button was used; in other words, they had given much more promise of survival. Post-mortem records showed better union without than with the button. There was probably a tendency to the development of stricture after the use of the button. This was, at any rate, markedly the case in gastro-jejunostomy. Hutchinson further remarked that primary resection in such cases, though naturally attended with a high mortality, yet offered the best chance of recovery. Of 11 cases treated by other so-called conservative methods—formation of an artificial anus, &c.—every one had died. This agreed with the results brought forward by other surgeons. It was nearly always advisable to perform the resection through a median abdominal wound. It was probable that the administration of an aperient immediately after the operation would lead to better results. Hutchinson stated he had operated on 140 cases of strangulated hernia with a total mortality of 25 per cent. A. E. Barker, in the discussion which followed, mentioned two successful cases of his own in which simple sutures had been used, and an unsuccessful one which the weight of a Murphy button had kinked the bowel and so caused obstruction. Marsh and Symonds both preferred suturing to using the button. A. H. Burgess[4] has also published a successful case of resection for this condition in a boy of 15. At the operation, when the sac was opened, there was a rush of fæculent fluid. The knuckle of small intestine two and a half inches in length was gangrenous, and had perforated at the summit of the loop. A second perforation was found on pulling down the entering bowel where the constriction had been. The wound was enlarged upwards, and four inches of intestine removed, and the bowel sutured with one continuous suture through the mucous membrane, and another continuous Lembert's suture through the sero-muscular coats. Recovery ensued without a bad symptom. The bowels were opened three times in the first 24 hours.

**Strangulated Hernia in an Infant.**—Charles MacLaurin[5] relates a case of this, the infant being only 14 days old. The hernia appeared as a tense, shining, œdematous mass of the size of a duck's egg, and had been irreducible 36 hours. There was no vomiting, but no motion had been passed for 24 hours. The sac contained four or five inches of congested intestine. This was returned after twice nicking the neck of the sac. The editors of the *Lancet* remark on the rarity of strangulation in infants, this no doubt being due to the softness of the structures at the neck of the sac. To relieve these cases it is recommended to raise the pelvis high on a pillow, the child lying on its back. Edmund Owen mentions a case in which he successfully reduced one by suspending a child by the feet, the top of the head just resting on the bed. The boy slept comfortably in this position, and the bowel quickly went back.

[3] Brit. Med. Jour., Feb., 1900. [4] Lancet, Feb., 1900. [5] Lancet, May, 1900.

